# Health Status of Sand Flathead (*Platycephalus bassensis*), Inhabiting an Industrialised and Urbanised Embayment, Port Phillip Bay, Victoria as Measured by Biomarkers of Exposure and Effects

**DOI:** 10.1371/journal.pone.0164257

**Published:** 2016-10-06

**Authors:** Jarrad K. Baker, Sara M. Long, Kathryn L. Hassell, Vincent J. Pettigrove, Marthe M. Gagnon

**Affiliations:** 1 Department of Environment & Agriculture, Curtin University, Bentley, Western Australia, Australia, 6102; 2 Centre for Aquatic Pollution Identification and Management (CAPIM), Bio21 Molecular Science and Biotechnology Institute, The University of Melbourne, Parkville, Victoria, Australia, 3010; 3 Centre for Aquatic Pollution Identification and Management (CAPIM), The University of Melbourne, Parkville, Victoria, Australia, 3010; Glasgow Caledonian University, UNITED KINGDOM

## Abstract

Port Phillip Bay, Australia, is a large semi-closed bay with over four million people living in its catchment basin. The Bay receives waters from the Yarra River which drains the city of Melbourne, as well as receiving the discharges of sewage treatment plants and petrochemical and agricultural chemicals. A 1999 study demonstrated that fish inhabiting Port Phillip Bay showed signs of effects related to pollutant exposure despite pollution management practices having been implemented for over a decade. To assess the current health status of the fish inhabiting the Bay, a follow up survey was conducted in 2015. A suite of biomarkers of exposure and effects were measured to determine the health status of Port Phillip Bay sand flathead (*Platycephalus bassensis*), namely ethoxyresorufin-*O-*deethylase (EROD) activity, polycyclic aromatic hydrocarbons (PAH) biliary metabolites, carboxylesterase activity (CbE) and DNA damage (8-oxo-dG). The reduction in EROD activity in the present study suggests a decline in the presence of EROD activity-inducing chemicals within the Bay since the 1990s. Fish collected in the most industrialised/urbanised sites did not display higher PAH metabolite levels than those in less developed areas of the Bay. Ratios of PAH biliary metabolite types were used to indicate PAH contaminant origin. Ratios indicated fish collected at Corio Bay and Hobsons Bay were subjected to increased low molecular weight hydrocarbons of petrogenic origin, likely attributed to the close proximity of these sites to oil refineries, compared to PAH biliary metabolites in fish from Geelong Arm and Mordialloc. Quantification of DNA damage indicated a localised effect of exposure to pollutants, with a 10-fold higher DNA damage level in fish sampled from the industrial site of Corio Bay relative to the less developed site of Sorrento. Overall, integration of biomarkers by multivariate analysis indicated that the health of fish collected in industrialised areas was compromised, with biologically significant biomarkers of effects (LSI, CF and DNA damage) discriminating between individuals collected in industrialised areas from observations made in fish collected in less developed areas of the Bay.

## Introduction

Located on the central south coast of Victoria, Australia, Port Phillip Bay is a large urbanised marine embayment encompassing an area of roughly 1950 km^2^ with a coastline approximately 264 km in length. The Bay is connected to Bass Strait through a narrow entrance which restricts average tidal movement, resulting in a flushing time of ~*ca* 12 months. The Bay is considered relatively shallow for its size with nearly half less than 8 m deep. [1]. The states’ largest city, Melbourne (population > 4 million), is located at the northern end of the Bay and the city of Geelong (population > 140,000) at the westernmost point. Melbourne is home to the busiest container shipping port in Australia; hence shipping traffic through the Bay is high. In 2008/2009, major dredging works were undertaken in the Bay to deepen shipping channels which raised major environmental concerns at the time. Due to the significant urbanisation and industrialisation of the catchment basin, introduction of contaminants into the Bay may potentially originate from a number of diffuse and point sources, including from the Yarra River which flows through Melbourne and discharges into the Bay. In order to preserve the social, economic and ecological values of the Bay, a number of pollution management measures were addressed by the late 1990s; such as the establishment of licencing discharges of urban and industrial effluents, the creation of management zones for specific purposes such as wildlife protection and increased sewering of catchments and diversion of industrial discharges into the sewerage system [2]. As a result of the shallow nature of Port Phillip Bay, of its restricted tidal exchange and of the expanding population living in the catchment basin, the potential for contaminant accumulation in the Bay ecosystem is considered high.

The coastline of the Bay has significant industrial and agricultural activity, including manufacturing plants and oil refineries. Chemicals discharged from industrial or urban waterways into the Bay ecosystem may have adverse effects on exposed organisms. One species important especially to recreational fisheries is the southern sand flathead (*Platycephalus bassensis*). Sand flathead stocks in Port Phillip Bay declined by 80–90% between 2000 and 2010, the reasons for which are generally unknown [3]. Previous studies have shown the southern sand flathead to be a suitable bioindicator species for health assessments of fish chronically exposed to low-level contaminants in the Bay [4]. Sand flathead have a sedentary, non-migratory lifestyle, are positioned high in the food chain [5], and are easily caught throughout Port Phillip Bay.

Biochemical markers (biomarkers) of fish health can inform on the health status of fish exposed to contaminants. One such commonly used biomarker is the hepatic microsomal ethoxyresorufin-*O-*deethylase (EROD) activity, which has been demonstrated to be a reliable indicator of exposure of fish to certain classes of organic pollutants. EROD activity is known to be induced by a range of pollutants including polycyclic aromatic hydrocarbons (PAHs), polychlorinated dibenzo-*p*-dioxins (PCDDs), polychlorinated dibenzofurans (PCDFs) [6] and polychlorinated biphenyls (PCBs) [7], all of which having been identified in the sediments of the Bay in the past [1]. Furthermore, previous studies measuring hepatic EROD activity in Port Phillip Bay sand flathead also indicated elevated EROD activity where fish were captured in the vicinity of high industrialisation/urbanisation [4, 8].

PAHs are potentially introduced to the Bay through industrial discharge/runoff, urban runoff and atmospheric fallout. In fish, metabolites of PAHs are produced by the liver following enzymatic oxidation and conjugation of free PAH, then predominantly expelled via the bile within a few days [9]. Measurement of biliary metabolites is considered an extremely sensitive marker of exposure to certain classes of PAHs, namely petroleum compounds [10–14]. Sand flathead captured in 1999 displayed a spatial variation in measured biliary PAH metabolites within Port Phillip Bay; with fish inhabiting areas of high industrialisation/urbanisation having significantly elevated metabolite levels compared to those from under developed areas [4]. Furthermore, ratios of lower and higher molecular weight PAH metabolites can be used to suggest the origin of PAH contaminant exposure [10, 15].

A variety of environmental pollutants have the potential to cause oxidative stress which may lead to damaged DNA molecules through free radical and reactive oxygen species (ROS) mechanisms [16]. One of the major products of these DNA damage mechanisms is the 8-oxo-7-hydro-2’-deoxyguanosine (8-oxo-dG) DNA lesion. Quantification of this oxidative DNA product is considered a biomarker of effect as it represents a biologically relevant adverse effect on the organism [17–20].

Carboxylesterases (CbE) are hepatic enzymes which play a significant role in the metabolism and subsequent detoxification of many agrochemicals and pharmaceuticals [21]. Studies assessing the inhibition of CbE activity in fish as a biomarker tool for environmental monitoring have found it to be a reliable biomarker of exposure to organophosphate pesticides (OPs) and carbamate insecticides in a number of species [21, 22]; however others have noted that inhibition of CbE activity is species and chemical specific [23]. No studies have been undertaken using CbE activity in sand flathead, or any closely related species; therefore this study will be the first to assess activity rates and discuss the potential use of this biomarker of exposure in environmental monitoring of demersal marine fish.

This study aimed to document the current health status of sand flathead inhabiting Port Phillip Bay. Sand flathead were collected at six sites throughout Port Phillip Bay and a suite of biomarkers of fish health were measured, along with complementary physiological indices. The following biomarkers were assessed: EROD activity, biliary PAH metabolites, DNA damage and CbE activity. Furthermore, ratios of biliary PAH metabolites were used to suggest the origin of hydrocarbon exposure. Information collected is compared both spatially, for inter-site variability, and where possible, temporally to previous studies undertaken on Port Phillip Bay sand flathead. Relevance of biomarkers that have not previously been measured in flathead are discussed; along with statistical methods used to interpret the wide range of biomarker data informing on the overall health of the fish collected at the six sampling sites. This study was successful in discriminating the health status of fish according to their area of origin, based on the integration of biomarkers of fish health.

## Materials and Methods

### Sample Collection and Biomarker Assays

**Fig 1:**
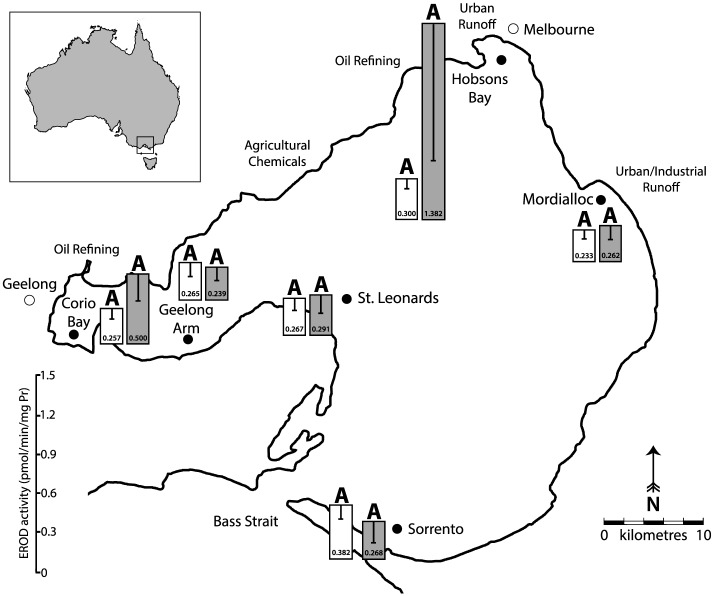
EROD activity levels (pmol/min/mg protein) in livers of male (filled bars) and female (white bars) sand flathead (*Platycephalus bassensis*) collected at six locations in Port Phillip Bay in 2015. Letters indicate statistical significance (α = 0.05) between sites, sexes are considered separately. Results are presented as antilog of geometric mean with 95% confidence interval about the geometric mean.

A total of 96 southern sand flathead were collected in February 2015 from six locations within Port Phillip Bay (Fig 1). Samples were collected by hook and line as directed under general research permit number RP1216 granted by the Department of Environment and Primary Industries (Victoria State Government). This study was specifically approved by the Curtin University Animal Ethics Committee under the approval number AEC_2015_05.

Fish were kept alive in aerated tanks during transport to the laboratory, until sacrificed humanely using the iki jime method [24, 25] prior to biopsies being collected. Fish were measured (standard length) and weighed, and a blood sample was collected from the caudal artery using a vacutainer. Blood samples were allowed to clot on ice for approximately 30 mins before being centrifuged at 5000 rpm for 10 min. The serum was isolated and immediately frozen in liquid nitrogen until 8-oxo-dG quantification by enzyme-linked immunosorbent assay (ELISA) as per methods described in Trevigen kit [26]. Following collection of all biopsies, carcass weight (body weight minus all viscera) was recorded. Sagittal otoliths were removed and cleaned for age determination by Fish Ageing Services (Queenscliff, Victoria).

Bile was extracted from each sample using a 1 ml syringe and immediately frozen in liquid nitrogen until biliary metabolite determination through semi-quantitative fixed fluorescence (FF) measurement [11, 13]. The method reports metabolized PAHs as ‘type of metabolites’. The expression type of metabolites refers to the various aromatic compounds, most occurring as conjugated metabolites of PAHs [27], that are measured in the bile at naphthalene-, phenanthrene-, pyrene or benzo(a)pyrene (B(a)P) specific excitation/emission wavelengths. Bile samples were diluted 1:500 using a 50% MeOH/50% H_2_O solution for pyrene and B(a)P fixed wavelength fluorescence and 1:2000 for naphthalene and phenanthrene fixed wavelength fluorescence. Protein content of diluted bile samples (bile:water 1:10) was determined according to Bradford [28], using bovine serum albumin (BSA—Sigma) as a reference standard. All fluorescent readings were measured randomly and in duplicate, with a high degree of replication between duplicates, using a Perkin-Elmer LS45 Luminescence Spectrometer. Fluorescent readings for naphthalene-type metabolites were performed at excitation/emission 290/335 nm, using 1-napthol (Sigma) as a reference standard. Readings were made at 260/380 nm for phenanthrene-type metabolites using phenanthrene (Sigma) as a reference standard. Readings for both pyrene-type and B(a)P-type metabolites used 1-OH pyrene (also called pyrenol-1, Sigma) as a reference standard, but differed in excitation/emission wavelengths with pyrene read at 340/380 nm and B(a)P read at 380/430 nm. A 10 nm slit width was used for both excitation and emission. Naphthalene-type metabolites are reported in μg of 1-naphthol fluorescence units equivalent per mg biliary proteins. Phenanthrene-type metabolites are reported in ng of phenanthrene fluorescence units equivalent per mg biliary proteins. Pyrene-type and B(a)P-type metabolites are reported in ng of 1-OH pyrene fluorescence units equivalent per mg biliary proteins. Concentrations of biliary metabolites are reported on the basis of milligrams of biliary protein because such a normalization can, to a large extent, account for changes in the level of fluorescent aromatic compounds due to the different feeding status of some fish [29] and due to variable water content of the bile [30]. Ratios of lower to higher molecular weight PAH biliary metabolites can be used as an indicator of the source of PAH contamination [10]. Phenanthrene-type biliary metabolites were chosen over naphthalene-type metabolites as the lower molecular weight compound given phenanthrene has a longer aquatic environmental half-life relative to naphthalene [31]. Ratios were calculated as phenanthrene-type biliary metabolites/B(a)P-type biliary metabolites.

EROD activity was assessed by the fluorimetric method described in Smith and Gagnon [7]. Briefly, livers were homogenized with a Diax 900 ultrasonic homogeniser and centrifuged at 12 000 g for 20 min. The post-mitochondrial supernatant (PMS) was collected and used immediately for the EROD assay carried out at 20°C. The reaction mixture was composed of 1250 μl 0.1 M HEPES buffer; 10 μl of 1.28M MgSO_4_; 30 μl of 0.5 mM NADPH; 50 μl of 40 mg/ml bovine serum albumin (BSA); and 50 μl PMS fraction, kept on ice. The reaction was started with the addition of 20 μl of 0.12 mM ethoxyresorufin; after 2 min, the reaction was stopped by the addition of 2500 μl of HPLC-grade methanol. The protein precipitate resulting from the addition of methanol was spun down and the amount of resorufin produced was measured on a Perkin-Elmer LS45 Luminescence Spectrometer at excitation wavelength 530 nm and emission wavelength 585 nm. The amount of resorufin was calculated from a linear resorufin standard curve varying from 0.00 to 425.50 pM/10μl standard solution, readings were relative to a blank fluorescence. Protein content of the supernatant was determined according to Bradford [28], using bovine serum albumin (Sigma) as a reference standard. All assays were performed in duplicate. EROD activity is expressed as picomol of resorufin produced per minute per milligram protein (pmol/min/mg Pr) in the PMS fraction.

CbE activity was determined using modified methods from Fisher, Crane, et al. [32] & Liang, Cui, et al. [33]. Livers were briefly homogenised using a Diax 900 ultrasonic homogeniser, 1 part liver to 3.5 parts 0.02 M phosphate buffer containing 1% triton X-100 (pH 8.0). The resulting homogenate was further diluted using 0.2 M phosphate buffer (pH 6.0) at a ratio of 1:35 and centrifuged at 14 000 g for 4 mins at 4°C. Protein content was determined according to Bradford [28], using bovine serum albumin (Sigma) as a reference standard. For CbE activity measurement, supernatant was diluted using 0.2 M phosphate buffer to a ratio of 1:5 so that the absorbance readings during the reaction time were within the linear range of the Bio-Rad iMark Microplate Absorbance Reader. The reaction was initiated by loading samples (20μl), into a 96 well microplate and adding 190 μl of assay buffer (containing 0.6% Fast Blue RR Salt and 100 mM 1-naphthyl acetate in 0.2 M phosphate buffer). All samples and blanks were run in triplicate. Absorbance was measured using a kinetic protocol at 450 nm every 60 seconds for 10 minutes with intermittent shaking and an increase in absorbance was observed. After verification of response curve linearity, CbE activity was calculated using an extinction coefficient of 9.1, and expressed as nmol/min/mg Pr.

### Statistical Analyses

Biomarker data, with the exception of morphometric parameters, were transformed using (log_10_+1) calculation prior to statistical analysis to achieve normality and homogeneity of variance. For morphometric parameters, EROD activity, bile metabolite levels (including ratios), CbE activity and DNA damage, analysis of variance (ANOVA) was used to detect true differences among sampling sites [34]. For EROD activity only, sexes were treated separately for statistical analysis as presence of estradiol exerts a down-regulation effect on cytochrome P4501A expression (CYP1A) in fish [35]. For all other parameters, sex was not a significant factor (*p >* 0.05) in the measured response. Post-hoc Tukey HSD tests were completed on variables with significant differences between sites (α = 0.05). For data that were transformed prior to ANOVA, results are presented as the antilogarithm of the geometric mean (arithmetic mean of transformed data). However, because back-transformed standard deviations are uninterpretable, variance is represented by the back-transformed 95% confidence interval of the geometric mean [34]. All data transformations and statistical analyses were completed using IBM SPSS Version 22.

To simplify the structure of collected variables, data were subjected to factor analysis using Principal Components Analysis (PCA) with orthogonal Varimax rotation. Output from PCA was analysed to ensure data met assumptions of the test. The Kaiser-Mayer-Olkin measure of sampling adequacy was 0.685 indicating sufficiency for factor analysis [36]. Bartlett’s Test of Sphericity was highly significant (*p* < 0.001), indicating patterned relationships between the variables, making PCA a suitable analysis [37]. Correlation matrix was used to analyse the data due to differences in units between variables. Components were extracted using an eigenvalues greater than 1.0 rule. Factor scores for each extracted component were saved and subjected to ANOVA to detect true differences between sites.

## Results

The number of sand flathead captured at each station, with morphological parameters measured are presented in Table 1. Fish collected from Hobsons Bay were longer and heavier (*F*_*5*, 90_ = 4.950, *p* < 0.001 and *F*_*5*, 90_ = 4.387, *p* = 0.001; respectively) than those from Mordialloc, Sorrento and St. Leonards. Significant differences in both condition factor (CF) and liver somatic index (LSI) were detected between sampling sites (*F*_*5*, 90_ = 5.350, *p* < 0.001 and *F*_*5*, 89_ = 8.712, *p* < 0.001; respectively). Sand flathead collected from Sorrento and St. Leonards had lower body weight for their length (CF) than those from Mordialloc and Corio Bay (*p* < 0.05); while those from Hobsons Bay and Geelong Arm were comparable to all sites (Table 1). Liver somatic index (LSI) increased in the order of St. Leonards < Sorrento < Geelong Arm < Corio Bay < Mordialloc < Hobsons Bay; with fish collected from St. Leonards and Sorrento having significantly smaller livers for their size (LSI) compared to those from Corio Bay, Mordialloc and Hobsons Bay (Table 1). A significant difference (*p* < 0.05) in LSI was detected between male and female fish collected from both Mordialloc and Hobsons Bay; however no differences were detected at other sites and for other morphometric parameters, consequently all parameters are presented as combined sexes.

**Table 1:** Morphometric parameters and numbers of sand flathead captured at each sampling station in Port Phillip Bay. Superscript letters indicate homogeneous subsets for each variable (α = 0.05). All values are presented as means ± SEM.

	*N*^1^	Age (years)	Standard Length (mm)	Weight (g)	CF^2^	LSI^3^
Sorrento	18	2.1 ± 0.6^a^	218.1 ± 3.3^a^	91.4 ± 4.5^a^	0.869 ± 0.013^a^	0.991 ± 0.050^a^
Geelong Arm	12	2.8 ± 0.4^a^	231.0 ± 6.6^ab^	120.4 ± 11.9^ab^	0.945 ± 0.020^abc^	1.080 ± 0.068^ab^
St Leonards	13	2.0 ± 0.1^a^	212.0 ± 8.8^a^	85.5 ± 4.4^a^	0.889 ± 0.021^ab^	0.895 ± 0.060^a^
Corio Bay	17	2.5 ± 0.4^a^	230.4 ± 8.8^ab^	126.7 ± 15.5^ab^	0.982 ± 0.031^c^	1.439 ± 0.102^bc^
Mordialloc	18	3.0 ± 0.4^a^	209.7 ± 4.5^a^	92.9 ± 6.3^a^	0.981 ± 0.017^c^	1.503 ± 0.116^c^
Hobsons Bay	18	2.3 ± 0.3^a^	248.3 ± 9.4^b^	157.8 ± 23.1^b^	0.957 ± 0.019^bc^	1.555 ± 0.122^c^

^1^ Sorrento Female (F) = 16, Male (M) = 2; Geelong Arm F = 7, M = 5; St Leonards F = 9, M = 4; Corio Bay F = 12, M = 6; Mordialloc F = 12, M = 6, Hobsons Bay F = 17, M = 1.

^2^ CF calculated as (W/L^3^)*100.

^3^ LSI calculated as (liver weight/carcass weight)*100.

Significant differences in 8-oxo-dG levels (DNA damage) were detected between sampling sites (*F*_*5*, 89_ = 5.226, *p* < 0.001) with sand flathead collected from Sorrento having significantly lower DNA damage levels compared to Corio Bay, Hobsons Bay and Mordialloc; while Geelong Arm and St. Leonards were comparable to all sites (Table 2). Male fish from Corio Bay and Hobsons Bay had significantly higher EROD activity levels when compared to females from the same site (*p* = 0.031 and *p* = 0.015; respectively). Analysing the sexes separately, no significant differences in EROD activity were detected between sampling sites for either males or females (*F*_*5*, 18_ = 2.063, *p* = 0.118 and *F*_*5*, 66_ = 1.199, *p* = 0.319; respectively—Fig 1). Statistical differences in CbE activity rates were detected between sampling sites (*F*_*5*, 85_ = 3.231, *p* = 0.010), with fish sampled from Sorrento expressing lower CbE activity than those from Geelong Arm and Corio Bay; while CbE activity from St. Leonards, Mordialloc and Hobsons Bay were comparable to all other sites (Table 2).

**Table 2:** Biomarker levels measured in sand flathead collected at 6 sites in Port Philip Bay, Australia. Superscript letters indicate homogeneous subsets for each variable (α = 0.05). All values are presented as means ± 95% confidence interval about the geometric mean.

	DNA Damage (8-oxo-dG)	CbE Activity (nmol/min/mg Pr)	Naphthalene-type biliary metabolites^1^	Phenanthrene-type biliary metabolites^2^	Pyrene-type biliary metabolites^3^	Benzo(a)pyrene-type biliary metabolites^3^	Ratio Phenanthrene-type / B(a)P type biliary metabolites
Sorrento	46.1 ± 22.6^a^	173.3 ± 27.1^a^	3,058 ± 613^a^	11,127 ± 2,281^ab^	737 ± 150^b^	394 ± 71^a^	29.6 ± 2.2^ab^
Geelong Arm	165.1 ± 72.8^ab^	236.7 ± 35.6^b^	2,102 ± 518^a^	6,638 ± 1,672^a^	410 ± 103^a^	273 ± 94^a^	25.6 ± 2.2^a^
St Leonards	204.7 ± 61.2^ab^	184.1 ± 28.3^ab^	3,496 ± 863^a^	13,386 ± 3,372^b^	866 ± 217^b^	472 ± 127^a^	30.4 ± 4.7^ab^
Corio Bay	426.1 ± 127.8^b^	238.7 ± 21.4^b^	3,148 ± 650^a^	12,329 ± 2,601^b^	626 ± 131^ab^	333 ± 81^a^	37.6 ± 1.8^bc^
Mordialloc	306.3 ± 199.6^b^	194.2 ± 34.1^ab^	2,328 ± 512^a^	8,594 ± 1,931^ab^	636 ± 142^ab^	320 ± 60^a^	27.3 ± 1.2^a^
Hobsons Bay	262.8 ± 150.6^b^	194.6 ± 27.7^ab^	2,729 ± 533^a^	13,465 ± 2,687^b^	884 ± 175^b^	332 ± 69^a^	41.1 ±1.6^c^

^1^ μg of 1-naphthol fluorescence units equivalent per mg biliary protein

^2^ ng of phenanthrene fluorescence units equivalent per mg biliary protein

^3^ ng of 1-OH pyrene fluorescence units equivalent per mg biliary protein

For both naphthalene-type and benzo(a)pyrene-type biliary metabolite levels, no significant differences were detected across sampling sites (*F*_*5*, 79_ = 2.210, *p* = 0.061 and *F*_*5*, 79_ = 1.036, *p* = 0.403; respectively -Table 2). Inter-site significant differences were detected for phenanthrene and pyrene-type biliary metabolite levels (*F*_*5*, 79_ = 4.537, *p* = 0.001 and *F*_*5*, 79_ = 4.096, *p* = 0.002; respectively). Phenanthrene-type metabolite levels were higher at Corio Bay, Hobsons Bay and St. Leonards compared to Geelong Arm (*p* < 0.05); while Sorrento and Mordialloc were comparable to all sites (*p* > 0.05) (Table 2). A significant difference (*p* < 0.05) in phenanthrene-type biliary metabolites was detected between male and female fish collected from St. Leonards. No other significant differences were detected between males and females at any other site for all measured biliary PAH metabolites, consequently all PAH biliary metabolites are presented as combined sexes. Levels of pyrene-type biliary metabolites increased in the order of Geelong Arm < Corio Bay < Mordialloc < Sorrento < St. Leonards < Hobsons Bay; with significantly lower levels in fish collected from Geelong Arm when compared to Sorrento, St. Leonards and Hobsons Bay (*p* < 0.05), while Corio Bay and Mordialloc were comparable to all sites (*p* > 0.05—Table 2). Significant differences were detected in phenanthrene-type/B(a)P-type biliary metabolite ratios between sites (*F*_*5*, 79_ = 7.603, *p* < 0.001). Corio Bay and Hobsons Bay had significantly higher ratios of phenanthrene-type/B(a)P-type biliary metabolites than both Geelong Arm and Mordialloc (*p* < 0.05 –Table 2). Ratios of phenanthrene-type/B(a)P-type biliary metabolites from Sorrento and St. Leonards were similar to all sites (p > 0.05) with the exception of Hobsons Bay (*p* < 0.05 -Table 2).

EROD and CbE activities were removed for PCA as no adequate relationships with any other variable were observed in an initial pre-screening of a correlation matrix (no correlations > 0.3). Using an eigenvalue cut-off of 1.0, two components were derived explaining a total variance of 74.96%. Table 3 displays factor loadings and individual % of variance for each component after Varimax rotation using a significant factor criterion of 0.3.

**Table 3:** Factor loadings and individual % variance for derived components (eigenvalues > 1.0) issued from PCA analysis.

	Principal Component	
	1 (51.31%)	2 (23.65%)
Phenanthrene-type metabolites	.961	
Pyrene-type metabolites	.941	
Naphthalene-type metabolites	.931	
B(a)P-type metabolites	.938	
Liver Somatic Index		.837
Condition Factor		.731
8-oxo-dG (DNA damage)		.637
Eigenvalue	3.59	1.66

**Fig 2:**
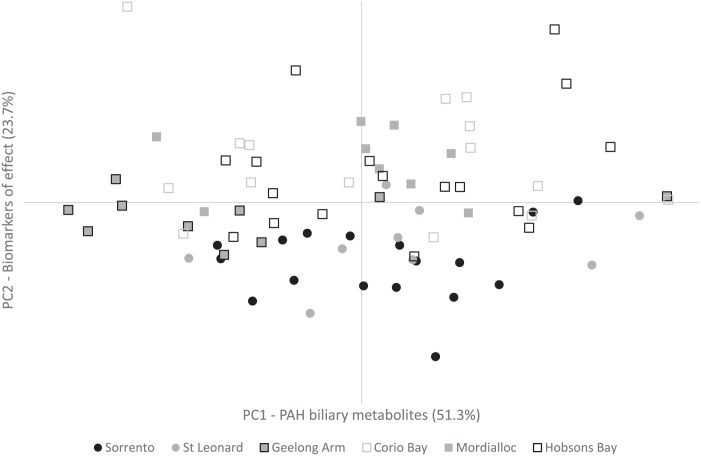
Principal Component Analysis plot. Principal Component 1 (biomarkers of exposure: biliary PAH metabolites) versus Principal Component 2 (biomarkers of effect: DNA damage, LSI and CF) provide a discrimination along Principal Component 2, between the low population density sites of Sorrento and St. Leonards (circles), and the highly industrialised/urbanised sites of Mordialloc, Hobson Bay and Corio Bay (squares).

Factor loadings show PC1 is composed of the four measured biliary metabolites or the ‘biomarkers of exposure’; while PC2 is composed of the physiological parameters LSI and CF, and DNA damage or the ‘biomarkers of effect’ (Table 3). ANOVA on the factor scores of each component highlighted significant differences between sites for both the biomarkers of exposure and the biomarkers of effect (*F*_*5*, 77_ = 2.807, *p* = 0.022 and *F*_*5*, 77_ = 11.477, *p* < 0.001; respectively). For the biomarkers of exposure, Geelong Arm and St. Leonards were the only sites with statistical differences between them (*p* < 0.05); with Geelong Arm having significantly lower factor scores. For the biomarkers of effect, Sorrento and St. Leonards had significantly lower factor scores than Mordialloc, Hobsons Bay and Corio Bay; while Geelong Arm was comparable to all sites except Corio Bay. Factor scores for the biomarkers of effect component increased as follows: Sorrento < St. Leonards < Geelong Arm < Mordialloc < Hobsons Bay < Corio Bay. An X-Y scatter of the two principal components factor scores with the low population density areas (Sorrento and St. Leonards) and highly urbanised/industrialised areas (Geelong Arm, Mordialloc, Hobsons Bay and Corio Bay) plotted separately, provides a visual discrimination of these areas along PC2 (Fig 2). ANOVA indicates factor scores between low population density and highly industrialised/urbanised sites as statistically significant for the biomarkers of effect component (PC2) (*F*_1, 81_ = 43.603, *p* < 0.001).

## Discussion

The aim of the present study was to evaluate the current health status of fish inhabiting Port Phillip Bay, Victoria using a suite of internationally recognised biomarkers of fish health. Minimal EROD activity in the sand flathead captured in 2015 at six locations in Port Phillip Bay is a strong indicator that exposure to EROD activity-inducing contaminants has reduced significantly since 1999 (the most recent previous studies assessing this biomarker in this fish species [4, 8]). Chemical analysis of the white muscle of sand flathead flesh further supports this, with levels of EROD activity-inducing chemicals (OCPs, PCBs & PAHs) below the limit of detection [38]. Gagnon and Holdway [4] suggested previous levels of EROD activity in Port Phillip Bay sand flathead were likely attributed to organic xenobiotics rather than exposure to PAHs, given that sites with elevated levels of PAH biliary metabolites did not all correlate with elevated EROD activity rates. The results from the current study suggest a reduction in the input of, a reduced bioavailability of, or a change in the type of organic trace pollutants entering the Port Phillip Bay ecosystem. This rationale is in line with other studies which observed a long-term declining trend in contaminant concentrations in various environmental compartments of the Bay, most likely a result of strong environmental regulations and of diversion of liquid waste to the sewerage system in the late 1990s [2].

Levels of naphthalene-type and B(a)P-type biliary metabolites in Port Phillip Bay sand flathead collected in 1999 showed a strong association with areas of high industrialisation and urbanisation [4]. The present study did not detect such a pattern in any of the measured PAH biliary metabolites. B(a)P-type biliary metabolite levels in 1999 sand flathead exhibited a 10 fold increase between the low population density area of St. Leonards and the highly industrialised site of Corio Bay. Levels of B(a)P-type metabolites in the present study were at the lower end of 1999 sampled sand flathead, although the semi-quantitative nature of the fixed fluorescence PAH biliary metabolite analysis method means comparisons between studies can be misleading [39]. When measured by the semi-quantitative fluorescence method, inter-site comparisons of biliary metabolite levels are more relevant than inter-study comparisons.

It has been suggested that ratios of petrogenic to pyrolytic PAHs may be used to indicate the source of PAH contamination [15]. PAHs of petrogenic origin are low molecular weight PAHs with two to three aromatic rings and are prevalent in unburned coals and fossil fuels; while the four to five ring PAHs of pyrolytic origin are formed during processes such as incomplete combustion of motor fuels and incomplete combustion of wood in forest fires and fireplaces [40]. Therefore ratios of low and high molecular weight PAH biliary metabolites can provide an indication of the source PAHs. Ratios presented in Table 2 indicate that fish collected in Corio Bay and Hobsons Bay are subjected to increased levels of PAHs of petrogenic origin compared to Geelong Arm and Mordialloc. This is likely attributed to the close proximity of both Corio Bay and Hobsons Bay to oil refineries (Viva Energy Geelong Refinery & ExxonMobil Altona North Refinery). Higher bile metabolite ratios observed at Corio Bay were significantly different to nearby Geelong Arm, suggesting the presence of the oil refinery has only a localised impact, which does not extend to Geelong arm, approximately 10 km away.

Hepatic metabolism of PAHs can lead to more biologically active PAH metabolites with highly carcinogenic properties [27, 41]. Toxicity of these metabolites comes from their ability to covalently bond to macromolecules such as proteins, DNA and RNA. Since fish do not have a highly developed DNA repair system [42], this may lead to many forms of lesions and adverse conditions in cells and in the organism; including mutagenesis, teratogenesis, and carcinogenesis [43]. Fixed fluorescence biliary metabolite analysis only provides information on exposure of organisms to PAHs and does not reflect biological effects this exposure may have. Therefore, biomarkers of exposure are often measured in conjunction with biomarkers of effects to provide a more comprehensive health assessment of the animals. In the present study, DNA damage through the quantification of 8-oxo-dG was used as a biologically relevant biomarker of effect.

There was a 6.1–9.9 fold increase in DNA damage between the low population density area of Sorrento and the highly industrial/urbanised sites of Corio Bay and Hobsons Bay (Table 2). The effectiveness of DNA repair systems in fish have been noted to be species dependent [42], and the relationship between detectable 8-oxo-dG levels and exposure to mixed contamination and duration of exposure are not currently known for sand flathead. Validation experiments using controlled exposures and durations of exposure of sand flathead to a variety of genotoxic chemicals are necessary before DNA damage in sand flathead can be used as an effective environmental monitoring tool. Nevertheless, DNA damage as measured by 8-oxo-dG in the serum of field-caught individual fish provides information on DNA alterations and informs on the risk associated with genotoxic chemicals in the aquatic environment. While DNA damage may prove a useful tool in environmental monitoring of genotoxic chemicals, it does not provide information on the chemical(s) responsible for the observed levels of DNA damage. Given that a wide range of environmental contaminants are known to be carcinogenic/mutagenic, DNA damage results alone do not provide enough weight of evidence for policymakers to make informed decisions e.g. prioritisation of sites for remediation. However, DNA damage results do provide a biologically significant physiological effect that has the potential to, if prolonged and severe, have detrimental impacts at the organism, population and potentially community levels [44], which might potentially have contributed to the decline in sand flathead populations observed in the past decade in Port Phillip Bay [3]. As a biomarker of effect, DNA damage is a significant contributor to the ecotoxicological toolbox available, however it has to be considered as an element in a suite of biomarkers to provide a more comprehensive picture rather than an isolated effect.

In this study, CbE activity was used as a biomarker of health in sand flathead for the first time in this species. Inhibition of CbE activity may be caused through interaction with many common agrochemicals such as pyrethroids, carbamates and organophosphates (OPs), along with some pharmaceuticals [21]. As Port Phillip Bay receives runoff from both agricultural and urban sources, there is the potential for CbE inhibition in biota inhabiting the Bay. The methodologies were successfully optimised for this species and significant differences in CbE activity were detected between sampling sites. Considering there were no detectable levels of CbE-inhibiting OPs in fish collected during a 2009 study of the lower Yarra River (one of the major rivers flowing into Port Phillip Bay) [45], and the magnitude of change between the sites lies within the range of 1.4 times, it is suggested the CbE activities detected in the present study are unlikely to be inhibited by OPs. Results did indicate CbE inhibition in fish collected from Sorrento when compared to Geelong Arm and Corio Bay, however the causal compound(s) for this difference are unable to be identified. The use of CbE activity as a biomarker of health in sand flathead requires further research using controlled exposure laboratory experiments to confirm baseline activity in healthy specimens and CbE inhibition by specific compounds.

In general, biomarkers interpreted on a ‘biomarker by biomarker’ basis points to a likely reduction in bioavailable contaminants in sand flathead since previous studies, mostly shown through negligible EROD activity and PAH biliary metabolites, but the inconsistency of other biomarkers, namely DNA damage and CbE activity make it difficult to report on current inter-site variability within the Bay. However, multivariate analysis provided some evidence that fish inhabiting urbanised/industrialised areas showed signs of compromised health over the fish captured in low population density areas. The most significant inter-site variability was explained by CF, LSI and DNA damage (Fig 2). The application of multivariate analysis for interpreting results of the present investigation is consistent with other studies which also found multivariate analysis to highlight links between variables that may not have been evident when individual biomarkers were considered in isolation [46, 47]. Overall, the results of the present study suggest that the health status of sand flathead inhabiting Port Philip Bay has improved since the last survey of 1999, as evidenced by lower levels of several biomarkers. Fish collected in non-industrialised or urbanised areas of the Bay (i.e. Sorrento) showed a better overall health status relative to those fish collected in highly urbanised/industrialised sites such as Corio Bay, Hobsons Bay and Mordialloc.

Pollution management measures implemented in late 1990s redirected industrial effluents to the sewer system and wastewater treatment plants, significantly reducing contaminant inputs into the Bay [2]. Along with other measures put in place two decades ago, such as a system of licensing discharges which improved the quality of urban and industrial effluents discharged into the Bay, and increased sewering of the catchment area [2], the pollution management of Port Phillip Bay appears to have been effective in reducing the contaminant loads bioavailable to sand flathead, and improved the health status of this iconic fish species.

## Conclusion

A range of biomarkers, both of exposure and of effects were used to determine the health status of Port Phillip Bay sand flathead. Temporal comparisons of EROD activity suggest a reduction of EROD activity inducing chemical exposure within the Bay since the 1990s. Levels of PAH biliary metabolites provided mixed results compared to previous studies; however, in general, the highly industrialised/urbanised sites did not display higher biliary metabolite levels as reported in earlier studies. Using ratios of metabolite types it was shown that Corio Bay and Hobsons Bay, each with nearby petrochemical processing, are enriched with petrogenic hydrocarbons. Quantification of DNA damage indicates a localised effect of exposure to pollutants to sand flathead, with a 10-fold difference between the industrial site of Corio Bay and the undeveloped site of Sorrento. This is further highlighted by the results of PCA which integrated DNA damage, CF and LSI into a single variable with significant differences between highly developed/industrialised and undeveloped areas of the Bay. CbE activity, a potential biomarker of exposure to organophosphate pesticides and other agricultural chemicals and pharmaceuticals was used for the first time in sand flathead. The test was optimised successfully for the species, however further research is required to confirm its prospective use in field studies. The present study highlights the value of using a range of biomarkers of fish health relevant to the expected contaminant mixture to be found in a typical urban embayment, such as Port Phillip Bay. Further to this, the integration of a suite of biomarkers in multivariate analysis provided a clearer interpretation of the current state of fish health in Port Phillip Bay.

## Supporting Information

S1 Dataset Physiological and biochemical markers measured in sand flathead (*Platycephalus bassensis*) collected in Port Phillip Bay in 2015. (PDF)
