# Supplementary material for: Health Status of Sand Flathead (Platycephalus bassensis), Inhabiting an Industrialised and Urbanised Embayment, Port Phillip Bay, Victoria as Measured by Biomarkers of Exposure and Effects
Source: PLoS One. 2016 Oct 6;11(10):e0164257. doi: 10.1371/journal.pone.0164257 (PMC5053506; doi:10.1371/journal.pone.0164257)
Supplement: S1 Dataset — (PDF) [file pone.0164257.s001.pdf]

FISHID	Site	Sex	Tot Length (mm)	Std Length (mm)	Body weight (g)	Liver weight (g)	Gonad weight (g)	Carcass weight (g)	**Age**	CF	LSI (Gutted)	GSI (Gutted)	BaP	Napthalene	Phenanthrene	Pyrene	DNA	CBE	EROD	Site
1	1	1	243	212	97.2	0.6	0.5	85.4	2	1.02	0.70	0.59	391.44	3133	12444	886.08	33.42	163	0.244	Sorrento
2	1	1	247	220	85.5	0.7	0.4	76.4	2	0.80	0.92	0.52	205.44	2005	7151	386.87	345.57	95	0.146	Sorrento
3	1	1	265	239	121.2	1.48	0.73	106	2	0.89	1.40	0.69	389.75	8076	22776	784.52	125.99	255	0.547	Sorrento
4	1	1	253	224	90.2	0.64	0.92	79.6	2	0.80	0.80	1.16	506.53	3565	12100	826.32	1.00	147	0.282	Sorrento
5	1	2	261	232	110.4	1.17	0.17	98.1	2	0.88	1.19	0.17	198.00	1886	7870	389.19	43.11	196	0.256	Sorrento
6	1	1	242	215	87	0.84	0.85	77.2	2	0.88	1.09	1.10	282.66	2666	8551	393.71	137.87		0.391	Sorrento
7	1	1	238	209	75	0.51	0.55	65.8	2	0.82	0.78	0.84	265.84	2455	6782	401.25	31.86	134	8.539	Sorrento
8	1	1	237	211	77.8	0.7	0.61	68.9	2	0.83	1.02	0.89	514.56	2828	9905	735.35	24.66	288	0.291	Sorrento
9	1	1	279	241	126.8	0.87	0.56	114.5	3	0.91	0.76	0.49	465.22	3338	15782	1060.54	74.45	155	0.228	Sorrento
10	1	1	207	184	52.2	0.35	0.26	45.3	2	0.84	0.77	0.57	355.55	1708	7955	637.11	95.59	248	0.208	Sorrento
11	1	1	247	219	91.7	0.92	0.68	81.4	2	0.87	1.13	0.84	284.75	2552	10515	793.81	137.87	145	0.335	Sorrento
12	1	1	236	205	79.8	0.75	0.51	69.2	2	0.93	1.08	0.74	309.55	2727	8710	494.80	82.91	192	0.721	Sorrento
13	1	1	251	225	94	0.81	0.62	85.1	2	0.83	0.95	0.73	441.06	3756	13253	1012.63	15.32	163	0.337	Sorrento
14	1	1	229	199	66	0.51	0.5	59.5	2	0.84	0.86	0.84	812.52	4852	16868	1161.90		87	0.287	Sorrento
15	1	2	240	215	83.9	0.77	0.08	75.7	2	0.84	1.02	0.11	353.96	2428	10196	829.62	16.37	206	0.281	Sorrento
16	1	1	252	220	94.7	0.98	0.61	86	2	0.89	1.14	0.71	498.01	2933	10044	965.37	23.20	156	0.24	Sorrento
17	1	1	256	222	92.9	0.68	1.06	83.6	2	0.85	0.81	1.27	683.27	4411	14177	927.62	50.86	242	0.331	Sorrento
18	1	1	268	234	119.9	1.53	0.93	107.1	2	0.94	1.43	0.87	689.39	5290	19232	1496.58	118.69	220	0.432	Sorrento
19	2	1	275	237	126.8	1.34	2.06	114.2	2	0.95	1.17	1.80	1211.17	7205	22121	1692.23	473.02	178	0.219	Geelong Arm
20	2	1	285	247	144.1	1.58	1.86	130.6	2	0.96	1.21	1.42	208.63	1259	4538	272.91	1018.10	206	0.346	Geelong Arm
21	2	1	259	225	100.5	0.87	0.63	90.7	1	0.88	0.96	0.69	344.48	1623	6184	590.07	228.83	187	0.496	Geelong Arm
22	2	2	238	207	89.4	0.89	0.67	82.3	5	1.01	1.08	0.81	209.68	1369	4643	284.96	113.49	198	0.258	Geelong Arm
23	2	1	258	225	102.9	1.44	0.98	91.6	3	0.90	1.57	1.07	360.17	3137	9981	735.04	143.37	230	0.24	Geelong Arm
24	2	2	225	198	71.3	0.57	0.13	63.8	1	0.92	0.89	0.20	461.38	2372	8386	682.90	21.40	266	0.228	Geelong Arm
25	2	2	227	199	77.1	0.74	0.27	70.8	5	0.98	1.05	0.38	268.03	2429	7328	336.39	164.66	211	0.277	Geelong Arm
26	2	1	260	226	95	0.96	2.17	85.1	2	0.82	1.13	2.55	257.15	1919	6204	382.89	128.07	224	0.192	Geelong Arm
27	2	2	263	229	117.2	1.11	0.86	106.9	5	0.98	1.04	0.80	#DIV/0!				204.00	236	0.217	Geelong Arm
28	2	1	284	249	134.7	1.43	1.73	122.3	2	0.87	1.17	1.41	139.52	1840	4196	200.01	177.89	227	0.258	Geelong Arm
29	2	1	309	270	211.4	1.39	5	192.7	2	1.07	0.72	2.59	137.86	1428	4477	243.57	110.23	441	0.204	Geelong Arm
30	2	2	299	260	174.1	1.24	1.35	157.3	4	0.99	0.79	0.86	160.99	2179	6761	377.74	126.44	333	0.22	Geelong Arm
31	3	1	214	188	52.8	0.39	0.2	47.3	1	0.79	0.82	0.42	#DIV/0!				129.13	257	0.181	St Leonards
32	3	1	241	212	81.2	0.68	1.17	72.8	2	0.85	0.93	1.61	435.59	2814	12517	872.72	121.75	149	0.234	St Leonards
33	3	1	233	201	77.1	0.53	0.68	68.6	2	0.95	0.77	0.99	445.77	3190	11135	762.20	182.03	229	0.208	St Leonards
34	3	1	241	209	72.8	0.67	1.07	65.9	2	0.80	1.02	1.62	#DIV/0!				136.73	182	0.246	St Leonards
35	3	2	226	189	67.3	0.6	0.03	61.8	2	1.00	0.97	0.05	#DIV/0!				392.16	158	0.27	St Leonards
36	3	2	243	212	83.9	0.63	0.07	76.1	2	0.88	0.83	0.09	992.40	8283	29723	2070.77	917.46	218	0.267	St Leonards
37	3	1	242	214	93.8	1.23	1.19	82.3	3	0.96	1.49	1.45	392.92	3126	11963	641.81	157.00	146	0.235	St Leonards
38	3	1	248	214	88.3	0.61	0.6	79.7	2	0.90	0.77	0.75	375.58	2417	8885	723.30	233.97	261	0.234	St Leonards
39	3	2	255	220	81.8	0.39	0.21	74.5	2	0.77	0.52	0.28	325.85	2471	8483	530.83	131.62	101	0.324	St Leonards
40	3	1	258	224	98.5	0.7	0.66	88	2	0.88	0.80	0.75	855.82	6110	18396	1341.14	120.25	153	1.691	St Leonards
41	3	1	254	219	105	0.9	0.75	95.2	2	1.00	0.95	0.79	476.46	3042	10672	1049.11	187.86	241	0.153	St Leonards
42	3	1	259	228	101.2	0.77	0.79	91.5	2	0.85	0.84	0.86	229.61	1491	5504	431.35	215.68	173	0.228	St Leonards
43	3	2	261	226	107.9	0.91	0.24	98.3	2	0.93	0.93	0.24	636.79	7108	45779	1157.86	332.96	209	0.309	St Leonards
44	4	1	274	238	138.7	1.98	1.71	127.5	2	1.03	1.55	1.34	510.25	3942	15163	929.98	347.63	272	0.405	Corio Bay
45	4	1	235	205	83.4	0.91	0.68	76.5	2	0.97	1.19	0.89	693.72	4137	21106	1095.04	503.75	227	0.262	Corio Bay
46	4	2	249	217	91.7	1.13	0.33	82.4	2	0.90	1.37	0.40	302.03	2817	10488	636.33	1018.10	197	0.097	Corio Bay
47	4	1	229	180	83.1	1.41	0.61	74.8	1	1.42	1.89	0.82	168.29	1440	5996	343.77	318.13	244	0.403	Corio Bay
48	4	1	316	284	225.4	2.8	4.56	202	6	0.98	1.39	2.26	199.72	2551	8039	438.27	160.96	221	0.157	Corio Bay
49	4	1	368	324	312.3	2.35	10.74	282.2		0.92	0.83	3.81	180.23	1791	5981	411.45	286.98	295	0.255	Corio Bay
50	4	2	273	239	143	2.57	1.07	129.7	6	1.05	1.98	0.82	402.06	3834	17314	734.25	703.71	235	0.317	Corio Bay
51	4	1	292	254	161	1.29	3.95	141.1	4	0.98	0.91	2.80	#DIV/0!				765.89	379	0.207	Corio Bay
52	4	2	217	189	60.3	0.62	0.2	55	1	0.89	1.13	0.36	#DIV/0!				168.90	228	1.034	Corio Bay
53	4	1	230	202	79.2	1.69	1.06	71	1	0.96	2.38	1.49	403.55	4981	15265	724.72	703.71	205	0.296	Corio Bay
54	4	2	229	200	83.6	1.38	0.39	74.8	2	1.05	1.84	0.52	398.34	4702	19256	754.76	218.38	284	0.41	Corio Bay
55	4	2	234	204	78.5	1.26	0.96	70.4	2	0.92	1.79	1.36	198.69	1934	8244	440.22	1018.10	244	0.381	Corio Bay
56	4	1	258	226	110.9	1.12	1.65	100.3	2	0.96	1.12	1.65	677.45	9655	36204	1435.88	268.10	218	0.284	Corio Bay
57	4	1	303	265	169.2	2.29	3.25	152.4	3	0.91	1.50	2.13	186.57	1607	6149	320.14	295.19	199	0.192	Corio Bay
58	4	1	250	222	90.2	0.98	0.93	81.2	2	0.82	1.21	1.15	560.07	5051	20872	911.61	1018.10	239	0.195	Corio Bay
59	4	1	274	236	118.7	1.05	1.32	106.9	2	0.90	0.98	1.23	474.75	3863	13365	715.40	164.33	265	0.287	Corio Bay
60	4	2	262	231	124.9	1.52	0.67	108.2	2	1.01	1.40	0.62	245.49	1861	9167	424.37	1018.10	173	3.141	Corio Bay
61	5	2	206	180	54.4	0.66	0.2	49.3	1	0.93	1.34	0.41	#DIV/0!				191.06		0.212	Corio Bay
62	5	2	214	186	58.1	0.8	1.31	51.9	2	0.90	1.54	2.52	#DIV/0!				703.71		0.307	Mordialloc
63	5	1	198	177	54.3	0.87	2.31	47.4	2	0.98	1.84	4.87	418.34	2785	9280	649.85	253.99		0.19	Mordialloc
64	5	2	218	191	74.5	0.68	3.48	65.8	4	1.07	1.03	5.29	#DIV/0!				187.90		0.326	Mordialloc
65	5	1	221	193	67.6	1.2	1.49	59.8	2	0.94	2.01	2.49	359.34	2480	9808	714.02	1371.52	225	0.341	Mordialloc
66	5	1	229	200	70.7	0.97	0.54	63.3	2	0.88	1.53	0.85	437.50	3033	11197	808.40	638.02	279	0.268	Mordialloc
67	5	1	230	201	88.9	2.15	3.16	74.1	2	1.09	2.90	4.26	#DIV/0!				238.94	223	0.291	Mordialloc
68	5	1	239	209	89.8	1.22	0.87	80.6	2	0.98	1.51	1.08	455.92	3431	13021	1064.61	703.71	206	0.287	Mordialloc
69	5	1	244	215	97	1.64	4.63	83.6	4	0.98	1.96	5.54	425.31	3240	10115	697.56	703.71	234	0.245	Mordialloc
70	5	1	254	219	95.2	1.46	1.65	83.8		0.91	1.74	1.97	358.02	2939	10318	738.01	392.57	185	0.279	Mordialloc
71	5	2	244	218	89	0.94	0.48	80.2	4	0.86	1.17	0.60	201.68	1875	6264	389.77	827.68	100	0.218	Mordialloc
72	5	2	247	215	99.1	0.75	2.95	87.9		1.00	0.85	3.36	496.53	3755	14730	1098.98	232.84	128	0.25	Mordialloc
73	5	1	258	224	118.5	1.48	1.08	106.8	2	1.05	1.39	1.01	199.77	1531	4997	374.68	1018.10	250	0.315	Mordialloc
74	5	2	252	220	99.6	0.78	3.83	87.6	7	0.94	0.89	4.37	308.78	2105	8371	691.45	150.55	345	0.277	Mordialloc
75	5	1	250	218	115.4	1.61	11.6	93.1	3	1.11	1.73	12.46	281.59	2243	7472	439.45	1018.10	187	0.117	Mordialloc
76	5	1	264	229	121.6	1.36	4.68	106.5	4	1.01	1.28	4.39	159.07	899	4105	292.55	703.71	146	0.127	Mordialloc
77	5	1	271	234	133.3	1.52	1.55	119.4	2	1.04	1.27	1.30	415.78	1998	8846	794.20		216	0.226	Mordialloc
78	5	1	279	245	144.4	1.35	1.45	127.8	5	0.98	1.06	1.13	226.58	2640	8921	792.67	1018.10	134	0.246	Mordialloc
79	6	1	301	265	168.2	2.61	2.26	151.4	2	0.90	1.72	1.49	291.97	2717	13325	808.80	225.63	202	0.335	Hobsons Bay
80	6	1	248	216	91.6	1.46	1.31	82.1	1	0.91	1.78	1.60	696.43	4068	24624	2112.32	1218.28	195	0.288	Hobsons Bay
81	6	1	227	199	73.9	0.58	0.55	67.6	1	0.94	0.86	0.81	611.81	3398	20593	1462.69	253.35	110	0.501	Hobsons Bay
82	6	1	274	239	123.7	1.05	0.69	116.3	1	0.91	0.90	0.59	208.87	1751	8053	502.97	194.55	178	0.258	Hobsons Bay
83	6	1	268	234	121.4	1.88	2.57	109.3	2	0.95	1.72	2.35	#DIV/0!				816.69	122	0.263	Hobsons Bay
84	6	1	259	224	105.3	1.43	1.93	94.4	2	0.94	1.51	2.04	320.68	2574	11807	772.73	952.41	179	0.291	Hobsons Bay
85	6	2	249	219	115	2.42	0.9	100.8	4	1.09	2.40	0.89	314.47	1618	8253	720.00	294.56	251	1.382	Hobsons Bay
86	6	1	256	222	103.7	1.32	1.09	93.2	2	0.95	1.42	1.17	203.08	1472	9520	669.33	1018.10	220	0.796	Hobsons Bay
87	6	1	288	249	166.2	1.91	9.65	143.2	2	1.08	1.33	6.74	169.61	2534	8055	409.50	134.48	187	0.282	Hobsons Bay
88	6	1	272	236	113.6	1.41	1.69	101.9	2	0.86	1.38	1.66	197.40	1951	8829	642.05	783.44	138	0.828	Hobsons Bay
89	6	1	258	224	98.6	1.44	1.4	88.7	2	0.88	1.62	1.58	356.21	3180	16679	978.30	296.01	342	0.123	Hobsons Bay
90	6	1	311	271	190.4	2.53	8.13	167.4	4	0.96	1.51	4.86	340.17	2934	15279	944.18	1.00	196	0.231	Hobsons Bay
91	6	1	285	248	146.4	1.84	1.58	130.8	3	0.96	1.41	1.21	458.91	2809	15706	1228.68	180.85	143	0.267	Hobsons Bay
92	6	1	290	254	156.8	1.53	2.35	142.5	2	0.96	1.07	1.65	264.44	2233	10433	750.96	143.67	248	0.326	Hobsons Bay
93	6	1	315	277	200.7	2.41	2.55	179.7	2	0.94	1.34	1.42	195.72	2486	9730	508.40	25.87	196	0.218	Hobsons Bay
94	6	1	290	251	185.3	3.85	7.49	162.5	3	1.17	2.37	4.61	563.05	4588	22381	1400.95	1018.10	180	0.233	Hobsons Bay
95	6	1	291	257	157.1	3.75	5.76	138.3	2	0.93	2.71	4.16	602.86	4140	22660	1309.78	703.71	225	0.31	Hobsons Bay
96	6	1	439	385	521.7	4.28	12.38	461.1	5	0.91	0.93	2.68	414.39	4707	21264	1258.60	753.26	353	0.191	Hobsons Bay
